# Improving the Outcome of Leukemia by Natural Killer Cell-Based Immunotherapeutic Strategies

**DOI:** 10.3389/fimmu.2014.00095

**Published:** 2014-03-17

**Authors:** Salem Chouaib, Gianfranco Pittari, Arash Nanbakhsh, Hanadi El Ayoubi, Sophie Amsellem, Jean-Henri Bourhis, Jan Spanholtz

**Affiliations:** ^1^INSERM U753, Institut de Cancérologie Gustave Roussy, Villejuif, France; ^2^Department of Medical Oncology, National Center for Cancer Care and Research, Hamad Medical Corporation, Doha, Qatar; ^3^Centre d’Investigation Clinique Biothérapies, Institut Gustave Roussy, Villejuif, France; ^4^Département d’Hématologie Clinique, Institut de Cancérologie Gustave Roussy, Villejuif, France; ^5^Glycostem Therapeutics, Hertogenbosch, Netherlands

**Keywords:** NK cells, NK-based immunotherapy, acute myeloid leukemia, NK cell expansion, hematopoietic stem cell transplantation

## Abstract

Blurring the boundary between innate and adaptive immune system, natural killer (NK) cells are widely recognized as potent anti-leukemia mediators. Alloreactive donor NK cells have been shown to improve the outcome of allogeneic stem-cell transplantation for leukemia. In addition, *in vivo* transfer of NK cells may soon reveal an important therapeutic tool for leukemia, if tolerance to NK-mediated anti-leukemia effects is overcome. This will require, at a minimum, the *ex vivo* generation of a clinically safe NK cell product containing adequate numbers of NK cells with robust anti-leukemia potential. Ideally, *ex vivo* generated NK cells should also have similar anti-leukemia potential in different patients, and be easy to obtain for convenient clinical scale-up. Moreover, optimal clinical protocols for NK therapy in leukemia and other cancers are still lacking. These and other issues are being currently addressed by multiple research groups. This review will first describe current laboratory NK cell expansion and differentiation techniques by separately addressing different NK cell sources. Subsequently, it will address the mechanisms known to be responsible for NK cell alloreactivity, as well as their clinical impact in the hematopoietic stem cells transplantation setting. Finally, it will briefly provide insight on past NK-based clinical trials.

## Introduction

Natural killer (NK) cells play a key role in the immune response to infections and malignancies by direct cytolysis of infected or transformed cells and by secretion of potent immune mediators. NK cell killing depends on the overall balance of inhibitory and activating signaling mediated by an array of surface receptors recognizing cognate ligands on putative targets. Besides their lytic activity, NK cells are cytokine-producing cells. Several reports highlight the fact that NK cells are also regulatory cells engaged in reciprocal interactions with dendritic cells, macrophages, T cells, and endothelial cells (1). NK cells have a central role in tumor-cell surveillance (2) as demonstrated in the setting of allogeneic hematopoietic stem cell transplantation (HSCT) (3–8).

Inhibitory NK receptors with specificity for HLA class I antigens are well described. Among these, killer Ig-like receptors (KIR) bind several HLA class I ligand groups (9), while the CD94–NKG2A/B heterodimer recognizes HLA-E (10). Several activating receptors are also described. Activating NKG2D is a major mediator of spontaneous cytotoxic activity. Natural cytotoxicity receptors (NCRs), including three molecules (i.e., NKp46, NKp30, and NKp44) specific for mostly unknown ligands (11, 12), mediate cell lysis of many cancer cells. Escape mechanisms from NK cell surveillance may involve NK cell quantitative deficiency and NK cell functional impairment.

Recent studies suggest that NK cell-based immunotherapy may continue to be an effective approach for patients with leukemia and emerging strategies are currently under investigation, initially based on adoptive transfer of NK cells.

This review will first describe current laboratory NK cell expansion and differentiation techniques by separately addressing different NK cell sources. Subsequently, it will address the mechanisms known to be responsible for NK cell alloreactivity.

## NK Cell Differentiation and Expansion

### NK cell expansion and NK cell generation methods

Successful NK cell immunotherapies require an adequate number of NK cells. In addition to NK cell number, NK cell purity and function are key factors for a clinically efficacious NK cell product (13). Various methods of NK cell expansion have been explored (14). In general, NK cells can be produced from peripheral blood (PB), umbilical cord blood (UCB), bone marrow (BM), embryonic stem cells (hESC), or induced pluripotent stem cells (iPCS). While short-term NK cell culturing does not normally enhance the functional capabilities for *in vivo* transferred NK cells, long-term expansion methods may yield large numbers of functional NK cells, which may potentially benefit cancer patients (15). Several alternative protocols for NK cell *ex vivo* expansion for adoptive immunotherapy have been reported to date. However, only some strategies have been developed under good manufacturing practice (GMP) conditions. In addition, substantial variability in NK cell expansion efficiency, phenotype, and function has been observed among different protocols and among individual donors (16–20).

### Expansion of NK cells for clinical purposes isolated from peripheral blood human

Several protocols for the expansion of PB NK cells are currently available, and others are under development. Various feeder cell-based systems have been used for NK cell expansion from peripheral blood mononuclear cells (PBMC), including third-party Epstein–Barr virus transformed lymphoblastoid B cell lines (EBV–BLCL), genetically modified K562 cells, or irradiated autologous cells (21–24). *Ex vivo* expansion of bulk peripheral NK cells using third-party EBV–BLCL feeders approximately yields a 180-fold NK cell expansion after 2 weeks of culture (22). Another expansion technique, yielding clinical valuable amounts of NK cells, is based upon K562 cell feeder double-transduced with IL-15 and 4-1BB (CD137) co-stimulatory ligand (K562–mb15–41BBL) (23). K562 cells transduced with IL-21 have also been used as feeder cells in NK co-culture systems (25). While K562–mb15–41BBL have been shown to expand and functionally enhance PB NK cells, K562 genetically engineered with membrane-bound IL-21 allow an even higher proliferation and cytotoxicity of expanded NK cells, which also display longer telomeres and less senescence (25). To expand CliniMACS-purified PB NK cells, autologous irradiated feeder cells have also been used as feeder cells in culturing systems containing human serum, IL-2, IL-15, and anti-CD3 antibody (21).

Many PB NK expansion strategies hold promise for NK-based immunotherapies. However, even using identical protocols, NK cell expansion yields and purity are typically inconsistent, and significant donor-to-donor variation is common. Moreover, complete absence of any residual viable tumor feeder in all final cell products is a critical requirement for large-scale NK cell therapy applications and their pharmaceutical translation.

The type of disposable cell culture systems for NK cell culturing also appears to influence the characteristics of the final cell product. Currently used disposable cell culture systems include flasks, bags, or WAVE^®^ bioreactors. Compared to flasks, use of bioreactors allow a 10-fold higher NK cell expansion after 3 weeks of culture (26), at the expense of a reduced purity of the final product, which also contains T cells (CD3+/CD56−) as well as NKT cells (CD3+/CD56+). Presence of T cells limits the application of this cell product to the autologous setting in the absence of downstream T-cell depletion.

### NK cell generation from umbilical cord blood

Umbilical cord blood is thought to be an excellent source for cell therapy applications. Initial work on positively selected cord blood NK cells, cultured on a feeder layer of mesenchymal stromal cells using a combination of IL-2, IL-15, Flt-3L, and IL-3, resulted in a mere 60-fold median expansion (27). In consideration of the low starting NK cell number in standard cord blood units, this approach is not feasible to generate NK cell numbers needed for a therapeutic NK cell product. Additionally, NK cell differentiation from CD34+ hematopoietic stem cells (HSC) has been addressed (28). Initially, research in this field focused on the *ex vivo* generation of NK cells from BM CD34+ cells (29–33), but later also involved CD34+ cells derived from UCB (34–39), a particularly rich source of HSC. These studies used different combinations of growth factor and cytokine mixtures, BM stroma cells, and culture media with or without animal or human sera. These culture systems generally contain components of animal origin; moreover, they fail to yield significant numbers of mature NK cells. For these reasons, it is unlikely that they will be used for clinical applications. Interestingly, the system reported by Silva et al. contained human serum without stroma cells and was therefore favored for clinical scaling-up (40). This NK cell expansion method was later abandoned, once stroma cells were recognized as a potential critical component for the enhancement of NK cell proliferation (40). In 2007, Kao et al. showed that serum-free expanded CD34+ cells may be differentiated into a NK cell product with an average purity of 40–60% after 5–7 weeks of culture, with a mean expansion rate of 300-fold. Such expansion, however, was obtained in the presence of fetal bovine serum (41). Later on, Vitale et al. studied the effects of methylprednisolone on CD34+ precursor cells and their ability to differentiate toward NK cells (38). They performed direct NK cell differentiation – i.e., no upfront CD34+ expansion – using RPMI-1640 medium supplemented with human serum, fetal bovine serum, and a cytokine cocktail including IL-15 and IL-21. At day 25, they observed a 10-fold NK cell expansion, with an average purity of approximately 30%.

In 2010, a novel cell culture technology for the *ex vivo* expansion and NK differentiation of UCB-derived CD34+ cells was developed. This technique was based upon a clinical-grade serum-free culture medium and a mixture of heparin and cytokines, in order to mimic the extra-cellular matrix BM microenvironment in the absence of feeder cells (42). This method yielded up to 10^10^ CD34+ cell-derived NK cells, generated in static cell culture bags and automated bioreactors. Importantly, NK cell products were not found to be contaminated by T cells, and may therefore be safely used in the allogeneic setting (43). Additionally, NK cells expressed high levels of activating receptors (e.g., NKG2D and NCR), and are able to efficiently kill myeloid leukemia and melanoma cell lines, as well as primary leukemia blasts. Furthermore, mouse studies have shown that they expand *in vivo*, initially home to the lung, and later to the blood, lung, spleen, liver, and BM and prevent outgrowth of local tumors in the BM (44). A phase I trial in elderly acute myeloid leukemia (AML) patients using NK cell products based upon this expansion technique is currently in progress (CCMO nr. NL31699 and Dutch Trial Register nr. 2818) (43).

Definitely, producing NK cells from CD34+ hematopoietic precursors can be practically advantageous, since stem cells can be isolated and frozen, and can overcome several obstacles posed by purified PB NK cells (15).

### NK cell generation from human embryonic stem cells or induced pluripotent stem cells

In contrast to the CD34+ UCB-derived NK cells, the generation of NK cells from embryonic stem cells or induced pluripotent stem cells is to date largely experimental (45). As previously mentioned, human NK cells can be differentiated from CD34+ HSC under certain culture conditions (31, 35, 42). Currently, optimizing the generation of CD34+ HSC from hESC and iPSC remains a major challenge to rationally approach hESC- and iPSCs-based NK cell therapy (45–48). hESCs or iPSCs derived CD34+ cells are known to be enriched for hematopoietic progenitors defined by colony-forming cells, which could serve as a suitable source for cell therapy (47). Some years ago, a method for the efficient generation of functional NK cells from hESC using a two-step procedure was described. NK cell products contained cytotoxic cells, displayed a mature NK cell phenotype, including CD16 and KIR, CD94/NKG2A, NKp46, NKp44, NKG2D, TRAIL, and FasL (49).

Engineering CD34+ cells, hESCs, or iPSCs to express chimeric antigen receptors (CAR) specific for tumor-associated antigens is a potentially promising immunotherapeutic strategy (48).

### NK cell lines for clinical use

Several authors have reported on the use of NK cell lines for therapeutic purposes. Unlike primary NK cells, whose *in vivo* life span is limited, NK cell lines are immortal. However, most NK lines are weakly cytotoxic to cancer targets. Conversely, the NK-92 cell line (Neukoplast, Conkwest, San Diego) has shown to mediate liable cytotoxicity and good expansion kinetics when cultured in bags or bioreactors. NK-92 has been well characterized in *in vitro* and shown to display remarkable anti-tumor activity in severe combined immunodeficiency (SCID) mouse xenotransplant models (50). Furthermore, it has been extensively tested in the clinical setting (51).

## Alteration of NK-Mediated Cytotoxic Response Against Leukemia

The escape of hematological malignancies from NK cell immunity can be explained by general mechanisms involving the quantitative deficiency of NK cells, their qualitative impairments caused by increased inhibition, decreased activation signaling, or by the negative influence of tumor microenvironment. It is widely recognized that leukemia cells may oppose a variety of immune escape strategies, including immune suppression and phenotypic mimicry, to elude NK-mediated killing.

The balance between activating and inhibitory signals received by NK cells will determine the NK cell-mediated elimination of leukemia cells. Decrease in ligands for activating receptors on leukemia cells could abolish NK cell dependent cytotoxicity. Furthermore, interaction of other ligands with cognate inhibitory receptors on NK cells surface could diminish NK cell killing activity, granule mobilization, and interferon production. Accumulating evidence indicates that chronic and acute leukemia cells could modulate NK cell activity by secreting soluble and exosomal ligands for NK cell receptors (52, 53). A key strategy used by leukemia cells to escape elimination by NK cells is related to apoptosis, and altered expression of molecules involved in different apoptosis signaling pathways may result in resistance to NK-mediated killing (54–56). In this setting, deregulation of the mitochondrial apoptotic machinery, functional blocks of caspase cascade, downregulation or inactivation of proapoptotic molecules, and upregulation of antiapoptotic molecules may all potentially favor leukemia chemoresistance or relapse. Finally, mechanism of NK cell resistance may also include drug resistance. For example, it has been shown that doxorubicin resistant cells increase expression of HLA class I on their surface (57).

## Capturing NK Cell Alloreactivity in Hematopoietic Stem Cell Transplantation

In the last 15 years, growing knowledge of NK tolerance to self, cancer immunosurveillance, and functional licensing has been extensively applied to HCT, in an effort to identify donors protecting from leukemia relapse through NK-based alloreactivity. Most studies investigating NK effects on transplantation outcome are rationally based upon one of the following three models: the missing-self recognition paradigm, the missing ligand model, and the activating receptor-based NK cell alloreactivity. This section addresses the mechanisms known to be responsible for NK cell alloreactivity, as well as their clinical impact in the HSCT.

### Recognition of HLA class I antigens by killer immunoglobulin-like receptors

The KIR gene cluster is mapped on chromosome 19q13.4, within the 1 Mb leukocyte receptor complex (LRC). It contains four ubiquitous “framework” genes (one centromeric, *KIR3DL3*; one telomeric, *KIR3DL2*; and two central, *KIR3DP1* and *KIR2DL4*) that flank centromeric and telomeric gene motifs characterized by extensive variation in both gene content (type and number) and polymorphism (58–60). Based on *KIR* gene content, multiple KIR haplotypes are identified, and categorized into two distinct groups, A and B. Group A haplotypes contain genes exclusively encoding inhibitory receptors and the activating *KIR2DS4*, while group B haplotypes contain genes encoding both inhibitory and activating receptors. Both KIR A and B haplotypes possess centromeric and telomeric gene content motifs. Products of functional KIR genes are type I transmembrane receptors with two (KIR2D) or three (KIR3D) highly homologous, extra-cellular immunoglobulin domains (61, 62, 63). Due to their clonal distribution in the NK repertoire, an individual NK cell may express one or more KIR (64, 65).

Inhibitory KIR may recruit the SH2-domain-containing tyrosine phosphatase 1 protein (SHP1) (66–69) through a single or double immunoreceptor tyrosine-based inhibitory motif (ITIM) contained in their long cytoplasmic tail (denoted L, i.e., KIR2DL; KIR3DL). Of several known inhibitory KIR, 2DL1, 2DL2, 2DL3, and 3DL1 are particularly relevant for HLA class I recognition. KIR2DL1 is specific for HLA-C2 group antigens (sharing the Asn^77^/Lys^80^ residues in the HLA-Cw heavy chain); KIR2DL2 and KIR2DL3 are specific for HLA-C1 group antigens (sharing the Ser^77^/Asn^80^ in the HLA-Cw heavy chain) (70, 71); and KIR3DL1 is specific for HLA-Bw4 ligands, sharing a group of sequence motifs in residues 77–83 of the heavy chain of certain HLA-B and HLA-A alleles (72–74).

Killer Ig-like receptor mediated activating signaling has also been identified. Unlike inhibitory KIR, they possess truncated portions that transduce activating signals via tyrosine phosphorylation of DAP12 and other proteins (75–77). Couples of cognate activating and inhibitory KIR, sharing almost complete homology (95–99%) in their extra-cellular domains, are recognized (75). Thus, activating KIR2DS1, KIR2DS2, and KIR3DS1 are, respectively, cognate receptors for the HLA class I-specific inhibitory KIR2DL1, KIR2DL2, and KIR3DL1. Counter-intuitively, the identification of natural ligands for activating receptors remains largely elusive. Ligands for activating KIR2DS2 and KIR3DS1 receptors have not been identified. While it cannot be excluded that these receptors recognize HLA class I/peptide complexes, current evidence indicates that they may not affect NK function by generating activating signaling when HLA-C1 and HLA-Bw4 are self-ligands. Unique among activating KIR, 2DS1 recognizes HLA-C2 group antigens, similar to its inhibitory homolog 2DL1.

### The missing-self recognition paradigm and NK cell alloreactivity

Inhibitory KIR interactions with cognate HLA class I ligands play a critical role in NK cell education and tolerance to self. In normal individuals, NK cells commonly possess KIR repertoires including one or more KIR with ligand specificity for self-HLA class I ligands (65). It is generally believed that such NK cells are rendered functionally competent, or licensed, by continuous signaling generated by inhibitory KIR upon interaction with self-HLA class I antigens (78, 79). HLA class I is critical to maintain NK tolerance to self, and cells failing to express sufficient levels of HLA class I ligands are promptly cleared by NK-mediated cytotoxicity. This phenomenon, known as missing-self recognition, was first postulated in a report by Kärre et al. describing that lack of MHC class I (H2) antigen expression rendered mice lymphoma cells highly sensitive to NK-mediated rejection (80).

Natural killer cells from donor-derived hematopoietic progenitor cells quickly reconstitute in HCT recipients (81–83). In the HSCT setting, HLA class I ligands of donor origin are believed to drive functional licensing. Reconstituted NK cells expressing one KIR for HLA class I present in the donor display stronger *in vitro* responsiveness than NK cells expressing one KIR for HLA class I present in the recipient, but absent in the donor (84). Additionally, CD107a externalization and IFNg production of NK cells reconstituting in recipients of donor unrelated or UCB grafts is markedly increased if they express KIR for donor self (85). This donor HLA-based NK education model implies, that the size of licensed donor NK cell is shaped by the frequency of inhibitory KIR-positive NK cells combined with the presence of cognate HLA class I ligands in the donor. Thus, KIR2DL1^+^ NK cells would acquire functional competence if donor is *HLA-C2*; KIR2DL2–3^+^ NK cells if donor is *HLA-C1*; and KIR3DL1^+^ NK cells if donor is positive for *HLA-A* or -*B* alleles possessing the Bw4 motif.

In HLA-mismatched HSCT, *HLA-C* allele groups (*C1* or *C2*), and/or the Bw4 epitope may be present in the donor and absent in the recipient. In this situation, the repertoire of licensed donor NK cells may include NK clones mediating missing-self allorecognition against host tissues. For example, KIR2DL1^+^/KIR2DL2–3^−^/KIR3DL1^−^clones from a *HLA-C2* positive donor may display allorecognition of missing self in a *HLA-C2* negative recipient. Recognition of missing self-HLA class I may improve the outcome of HSCT. T-cell-depleted haplotype-mismatched grafts from NK alloreactive donors mediate strong graft vs. leukemia (GvL) effects in AML recipients, allowing for lower risk of relapse and better survival (3, 4). While in haplotype-mismatched grafts, the effect of KIR-ligand incompatibility is well established, studies on mismatched unrelated HSCT reported conflicting results. In this setting, Giebel et al. showed that donor NK cell alloreactivity improves recipient survival (86). A protective effect of KIR-ligand incompatibility on post-transplantation relapse was later confirmed in myeloid malignancies (87) and multiple myeloma (88). However, most studies failed to show a beneficial effect of donor NK cell alloreactivity on the outcome of mismatched unrelated HSCT (89–92). Similarly, studies exploring the clinical impact of NK alloreactivity mediated by KIR-ligand mismatch in UCB grafts have yielded variable results (93, 94). These inconsistent observations are possibly influenced by complex variables, such as donor KIR genotype (95), disease category, type of conditioning T-cell depletion, post-transplantation immune suppression for graft vs. host disease (GvHD) prophylaxis. For example, T cells may dominate alloreactive phenomena in mismatched unrelated HSCT and counteract the clinical benefit of NK alloreactivity (91). Accordingly, *in vivo* T-cell depletion with anti-thymocyte globulin (ATG) has been shown (86, 88, 96) to enhance the favorable impact of NK cell alloreactivity on HSCT outcome.

### The missing ligand model and NK cell alloreactivity

Killer Ig-like receptors and HLA genes are mapped on different chromosomes, and segregate independently according to a Mendelian inheritance pattern. Therefore, certain individuals may have *KIR* genes but not the corresponding HLA/KIR-ligand groups (97). KIR receptors are clonally distributed on NK cell surface, allowing for the possibility, that subpopulations of NK cells exclusively express KIR with ligand specificity for non-self-HLA class I ligands. These NK cells are not classically licensed by self-HLA class I ligands during their development, and are believed to be hyporesponsive to stimulation in physiologic conditions. During post-transplant immune reconstitution, however, this non-licensed status may be transiently suspended during post-transplantation immune reconstitution, and effector functions could indeed be mediated by donor NK cells expressing KIR with ligand specificity for non-self-HLA class I. An important implication of the missing ligand model is that NK alloreactivity would be observed even in the absence of donor/recipient KIR-ligand mismatch, a necessary condition for missing self-mediated NK alloreactivity.

Several studies exploring the effect of the missing ligand model on HSCT outcome indicate, that donors possessing inhibitory KIR but not the corresponding HLA class I ligand do mediate beneficial NK effects in HLA-identical siblings or HLA-matched unrelated recipients (5, 98–100). Hsu et al. originally explored a cohort of 178 subjects receiving a T-cell-depleted graft from a HLA-identical sibling. In patients with AML and myelodysplastic syndromes (MDS), lack of one or more HLA ligand for donor KIR resulted in lower relapse and better survival (5). Following the identification of beneficial NK effects in the HLA-matched setting, the function of classically non-licensed NK cells has been directly explored in the context of post-transplantation reconstitution. In T-cell-depleted grafts from HLA-identical siblings, NK cells expressing KIR for non-self-HLA display strong IFNγ production and cytotoxicity to target stimulation during the first trimester post-transplantation (101). These findings have not been confirmed in a cohort of recipients of T cell-replete grafts from HLA-identical siblings. Here, reconstituted NK cells expressing KIR for non-self-HLA ligands displayed tolerance to self. Moreover, lack of self-HLA ligands for donor inhibitory KIR was found to have no effect on HSCT outcome (102). Presence or absence of T cells in the graft may differentially affect self-tolerance of non-licensed donor NK cells post-transplantation. Regardless, the interpretation of these conflicting results demands further studies on tolerance to self of donor NK cells reconstituting in the HLA-identical host.

### Activating receptor-based NK cell alloreactivity

Because ligands for most activating KIR are currently unknown, studies reporting associations between activating KIR and HSCT outcome are not generally supported by the identification of an underlying immunological background mechanistically explaining the observed NK-mediated alloreactivity. In a cohort of 65 graft recipients from HLA-identical siblings, donors with genotypes containing both *KIR2DS1* and *KIR2DS2* genes provided protection from relapse (103). Donor activating KIR was later found to control CMV reactivation post-transplantation. Recipients of T cell-replete grafts were found to have a remarkable reduction of the incidence of CMV reactivation, if donor possessed more than one activating *KIR* genes (104). Confirmation of the protective effect against CMV reactivation by donor activating *KIR* was concomitantly reported by another group (105).

In 2009, Cooley et al. investigated the effect of different donor KIR haplotypes in 448 AML recipients of unrelated T cell-replete HSCT. Recipients of KIR B/x grafts (i.e., homozygous or heterozygous for KIR B group haplotypes) displayed a higher 3-year-overall survival (6). In a cohort of 1086 AML recipients of unrelated grafts, the same group later compared the contribution to HSCT outcome of donor centromeric and telomeric group A and B KIR haplotypes. Donors homozygous for centromeric B gene content motifs (Cen B/B) most strongly associated with low risk of relapse and prolonged survival (7). Among activating KIR, activating KIR2DS2 is mapped on the centromeric region of several B group haplotypes, and may thus mediate the clinical benefit observed for Cen B/B donors through interaction with an unknown ligand expressed on leukemia cells (7).

Recent studies investigated the effect of telomeric activating *KIR3DS1* and *KIR2DS1* genes on transplantation outcome. Patients receiving unrelated grafts from *KIR3DS1* donors exhibited a lower risk for grade II–IV GvHD and mortality (8, 106). Activating KIR2DS1 is found in approximately 1/3 Caucasians, and commonly occurs in individuals positive for *HLA-C2* (*C1/C2*; *C2/C2*) (107–109). KIR2DS1 expression occurs in more than 20% NK cells (109), and 2DS1 single positive (KIR2DS1^SP^) NK cells (i.e., lacking inhibitory KIR expression), may also be identified. In *HLA-C2* individuals, KIR2DS1^SP^ NK cells may potentially display auto-reactivity to normal self-tissues. Compared with *HLA-C1* donors, KIR2DS1^SP^ NK cells from *HLA-C2* homozygous individuals are hyporesponsive to a HLA-C2 positive target cell (108). Similarly, mice studies described hyporesponsiveness of activating receptor-positive NK cells resulting from *in vivo* chronic interaction with a viral ligand (110, 111). Recently, KIR2DS1^SP^ NK clones displaying *in vitro* anti-HLA-C2 cytotoxicity have been identified in all *HLA-C* genotypes (112). In *C2:C2* individuals, these clones are significantly reduced in frequency. In contrast, anti-HLA-C2 reactive KIR2DS1^SP^ clones from *C1:C2* individuals are common, and functionally indistinguishable to those obtained from *HLA-C2* negative (*C1:C1*) donors (112). These observations indicate that tolerance development is affected by HLA-C2 ligand expression density, and are consistent with the “functional NK plasticity” phenomenon described in mice studies (113, 114). The effect of donor *KIR2DS1* on HSCT outcome has been assessed in a cohort of 1277 AML recipients of unrelated HLA-matched or 9/10 mismatched HSCT. Patients receiving grafts from *KIR2DS1* donors displayed a decreased rate of leukemia relapse (8). In agreement with experimental findings (112), *KIR2DS1*-mediated protection was observed in *HLA-C1* (*C1:C1* and *C1:C2*) donors, but not in *HLA-C2* homozygous donors, presumably due to the strong tolerogenic effect on KIR2DS1-positive NK cells mediated by the *HLA-C2* homozygous genotype (8).

## NK Cell Therapy Modalities

Early studies by Miller et al. in 2005 have open the way to demonstrate the safety of adoptive transferred human NK cells in patients with AML. Human haplo-identical NK cells could be expanded *in vivo* and transferred in patients with poor prognosis AML. Patients had to be prepared with an immunosuppressive high dose alkylating and purine analog conditioning regimen they can promote recirculation of infused allogeneic NK cells. Further haplo-identical allogeneic NK cells infusions were applied in patients with poor prognosis Hodgkin lymphoma and various solid tumors, i.e., melanoma (115) and renal cell carcinoma. In order to promote NK cell expansion, a lympho-depletion preparative regimen is required for the patient in all studies reported. To date, adoptive immunotherapy with unstimulated or IL-2 activated NK donor NK cells infusion is used for patients undergoing haplo-identical hematopoietic progenitor cells transplantation to prevent from relapse. No serious and immediate adverse effects have been observed following allogeneic NK cells infusion.

Clinical scale protocols to collect, enrich, and expand purified NK cells are nowadays feasible from cord blood, human donor apheresis, and dedicated cell lines. These cell therapy procedures remain costly and time consuming. They need an experimental lab with GMP facilities together with the skills and allowance to perform the cell manipulation.

The currently defined cell product has optimized T-cell depletion to avoid GvHD and allogeneic NK cell expansion; but we are still lacking a clinical standardized procedure for the use of enriched and expanded NK cells (116).

The most common limitation of the procedure remains the inability of collected, enriched, activated, and expanded allogeneic NK cells to expand properly *in vivo*. At first, a huge loss of NK cells during the isolation procedure can be seen following T-cell depletion procedure. Then CD56+/CD3− NK cells may be rejected by cytotoxic T cells or suppressed by myeloid-derived suppressor cells or Tregs.

To date, ongoing clinical scale protocols aim to both select CD56+/CD3− NK cells and increase drastically the total number of NK cells. Another limitation comes from the use of high dose alkylating chemotherapy-based protocols prior to infusion of NK cells that can increase the toxicity and efficacy of NK cell-based immunotherapy. Infusion of allogeneic NK cells can be performed following standard doses regimen together with immune modulation with steroids and IL-15.

In the field of BM transplantation, infusion of donor-derived stimulated and expanded CD56+/CD3− allogeneic NK cells may be used in repetitive injections to promote the GvL effect. Further studies have to be designed to enhance the NK cell therapy efficiency: improving the NK cell selection, processing, *ex vivo* expansion, better designing of lympho-depletion protocols for the patient and infusion rate of NK cells for a optimized control of residual leukemic cells.

## Conclusion

Natural killer cells continue to attract a lot of interest in transplantation. However defects in NK cell cytotoxicity have been observed in all hematological malignancies and the escape of hematological malignancies from NK cell immunity can be explained by general mechanisms. The latter are common to all immune-effector cells involving the quantitative deficiency of NK cells, their qualitative impairments caused by increased inhibition, or decreased activation signaling.

Natural killer cell selection from leukapheretic products is a largely inadequate approach to obtain large numbers of cancer-reactive NK cells. Given the clonal and stochastic distribution of activating and inhibitory receptors on NK cell surface, pools of cancer-reactive NK cells generally represent a minority of the bulk CD3^−^, CD56^+^ population. Several methods for *ex vivo* NK cell expansion have been attempted to date. However, none of the described approaches have definitively proved able to effectively circumvent the issue of limited NK cell availability and efficiency. Although an important anti-tumor role for alloreactive NK cells has been shown in patients with AML either after stem-cell transplantation or adoptive transfer of haplo-identical NK cells, their clinical efficacy in human trials has been modest, presumably due to tumor escape by alteration of NK cell function and resistance to killing. In this respect, the ability to manipulate not only the balance of activating and inhibitory receptors on NK cells but also their cognate ligands, as well as the sensitivity of tumor cells to apoptosis, opens new perspectives in NK cell-based immunotherapy. Sensitization of tumor cells to activated cytotoxic lymphocytes by up-regulating either TNF death receptors or effector-activating ligands on tumor cells combined with immunotherapy has been pursued to overcome tumor-cell resistance and establishes an effective anti-tumor response. Another therapeutic approach to enhance NK cell cytotoxicity is the use of cytokines, such as IL-2, IL-12, and IL-18. Chemokine manipulation is also of interest as molecules may both attract NK to the tumor-cell microenvironment and stimulate their cytotoxic properties. Another attractive approach to enhance NK cytotoxicity is to use monoclonal antibodies (mAbs). IPH-2101, a fully human IgG4 anti-KIR mAb (developed by Innate Pharma) is currently being tested in phase I and II clinical trials in patients with AML and multiple myeloma (117). Its blockade of inhibition could allow NK cell activation when activating ligands are present on target cells. It should also be emphasized that bispecific mAbs directed against both the target cells and NK cells are also currently under investigation. Anti-CD20 mAbs that have enhanced affinity for CD16 have been also developed, and they are more effective at NK activation than rituximab (118).

One limitation of the use of NK cells is due to the great capacity of leukemic cells to escape to NK recognition and killing. This resistance remains indeed a drawback in immunotherapy of hematological malignancies. In this regard, adoptive transfer of polyclonal or clonal NK cells with mismatch NK inhibitory receptors and HLA class I ligands would produce GvL (GvHD) in the absence of GvHD. The simultaneous differentiation of the effective killer effectors, the boosting of NK function, their expansion, and the sensitization of leukemic targets will offer potential opportunities in the treatment of hematological malignancies. Therefore, how to expand UCB stem cells for optimal downstream generation of terminally differentiated NK cells potentially alloreactive to leukemic cells is at present very challenging.

## Conflict of Interest Statement

The authors declare that the research was conducted in the absence of any commercial or financial relationships that could be construed as a potential conflict of interest.
